# Supplementary material for: A methodology for the design of experiments in computational intelligence with multiple regression models
Source: PeerJ. 2016 Dec 1;4:e2721. doi: 10.7717/peerj.2721 (PMC5136129; doi:10.7717/peerj.2721)
Supplement: Data S1 — Datailed results from UC Irvine Machine Learning Repository (Housing, Machine CPU, Wine Quality, Automobile and Parkinson) and the 3 Use Cases (Protein Corona, Gajewicz Metal Oxides and Aquatic Toxicity) [file peerj-04-2721-s001.zip › 6_UseCases/3_AquaticToxicity/PLS.details.csv.repeatedcv.split8.pdf]

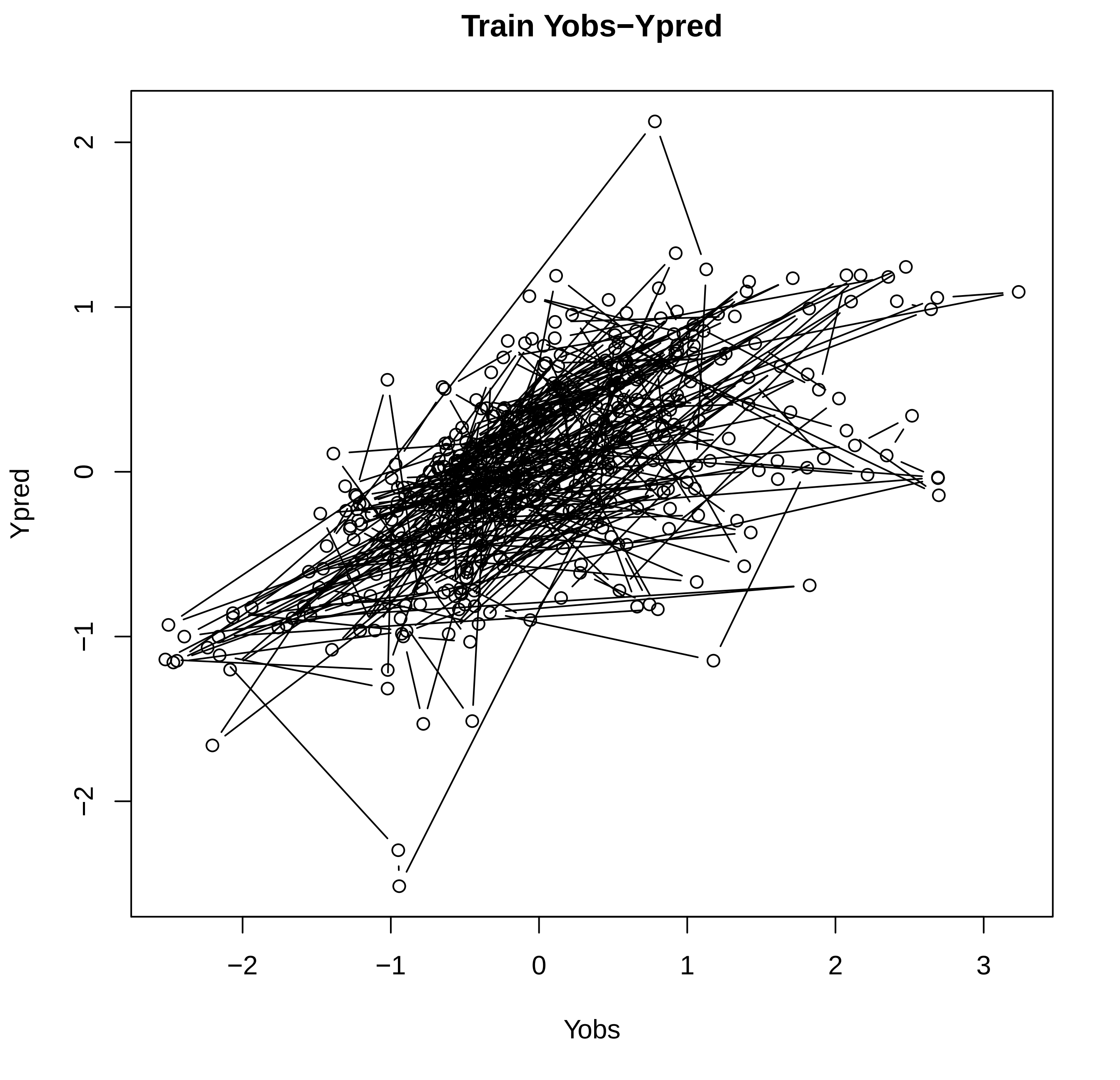

Train Yobs−Ypred
Ypred
Yobs
−2
−1
0
1
2
3

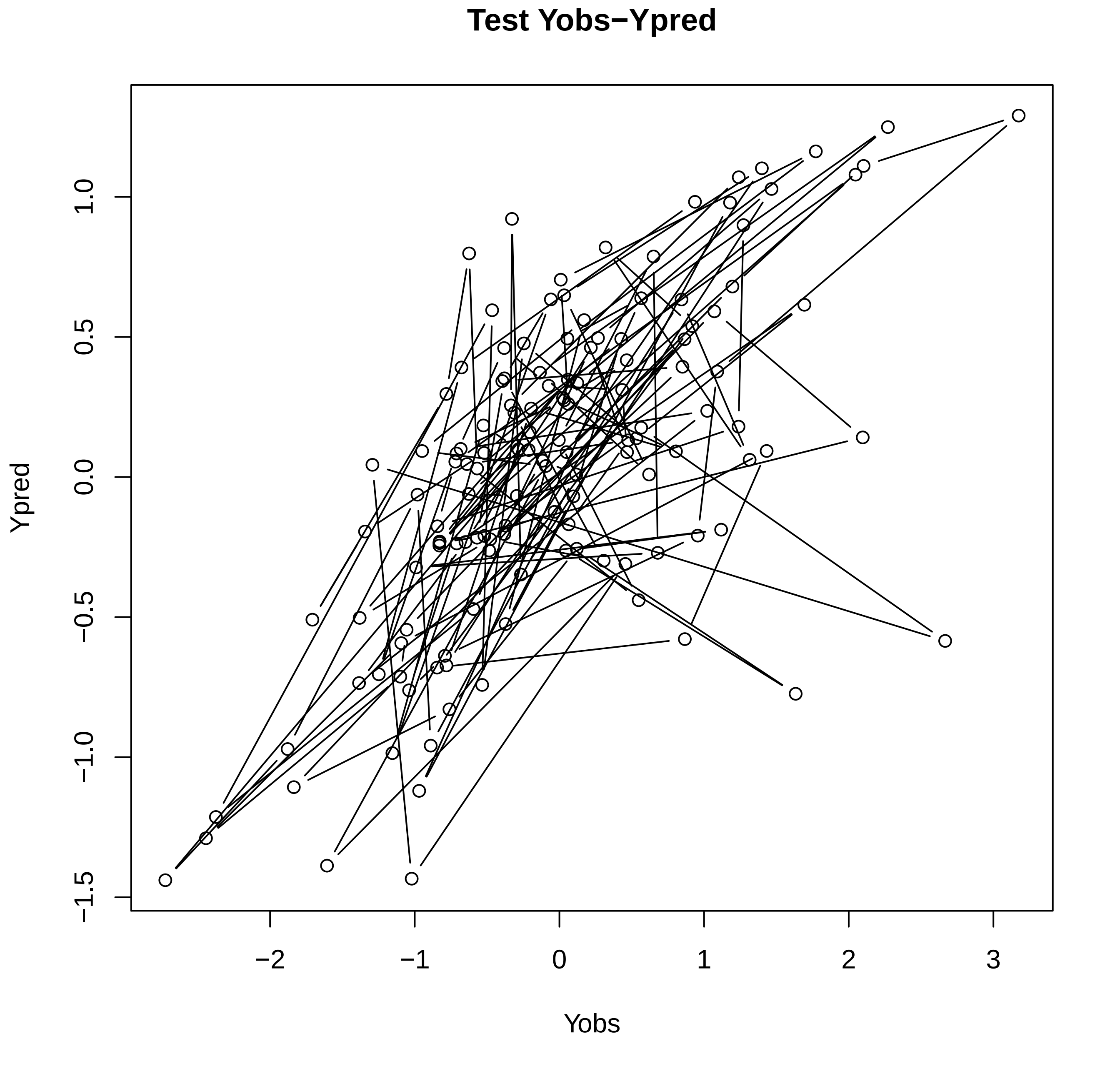

Test Yobs−Ypred
Ypred
Yobs
1.0
0.5
0.0
−0.5
−1.0
−1.5
−2
−1
0
1
2
3

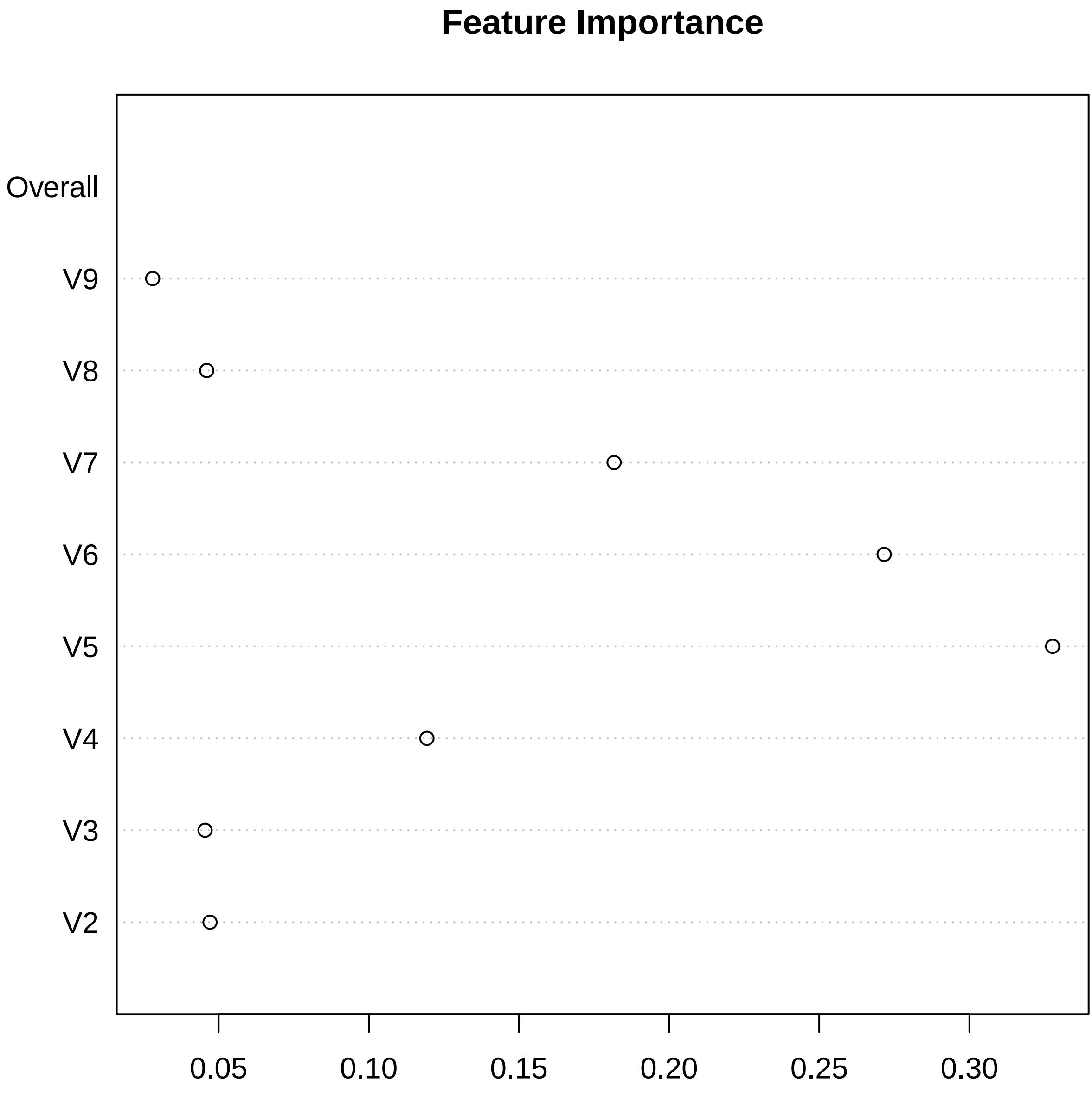
Feature Importance
Overall
V9
V8
V7
V6
V5
V4
V3
V2
0.05
0.10
0.15
0.20
0.25
0.30

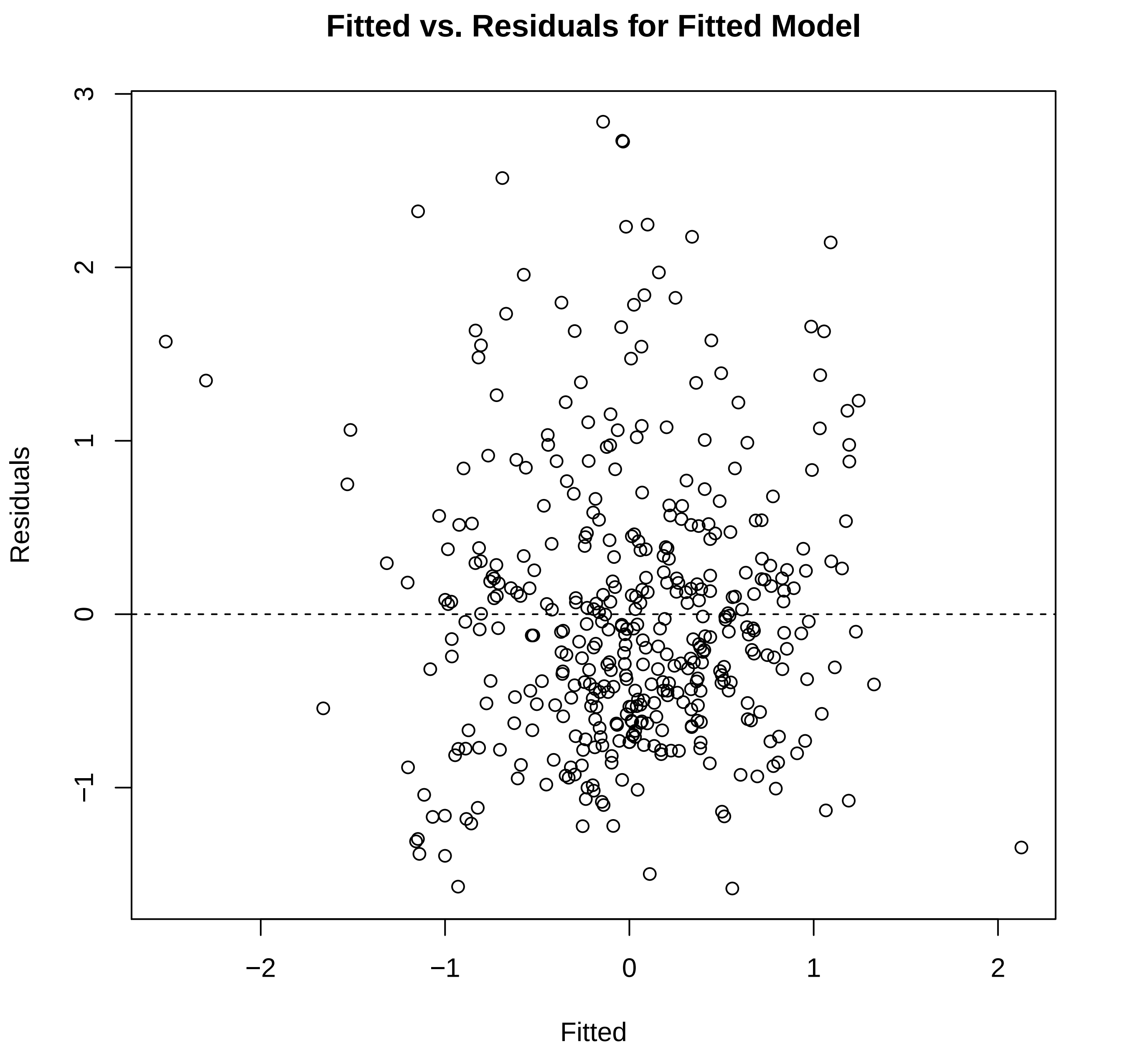
Fitted vs. Residuals for Fitted Model
Residuals
3
2
1
0
−1
−2
−1
0
1
2
Fitted

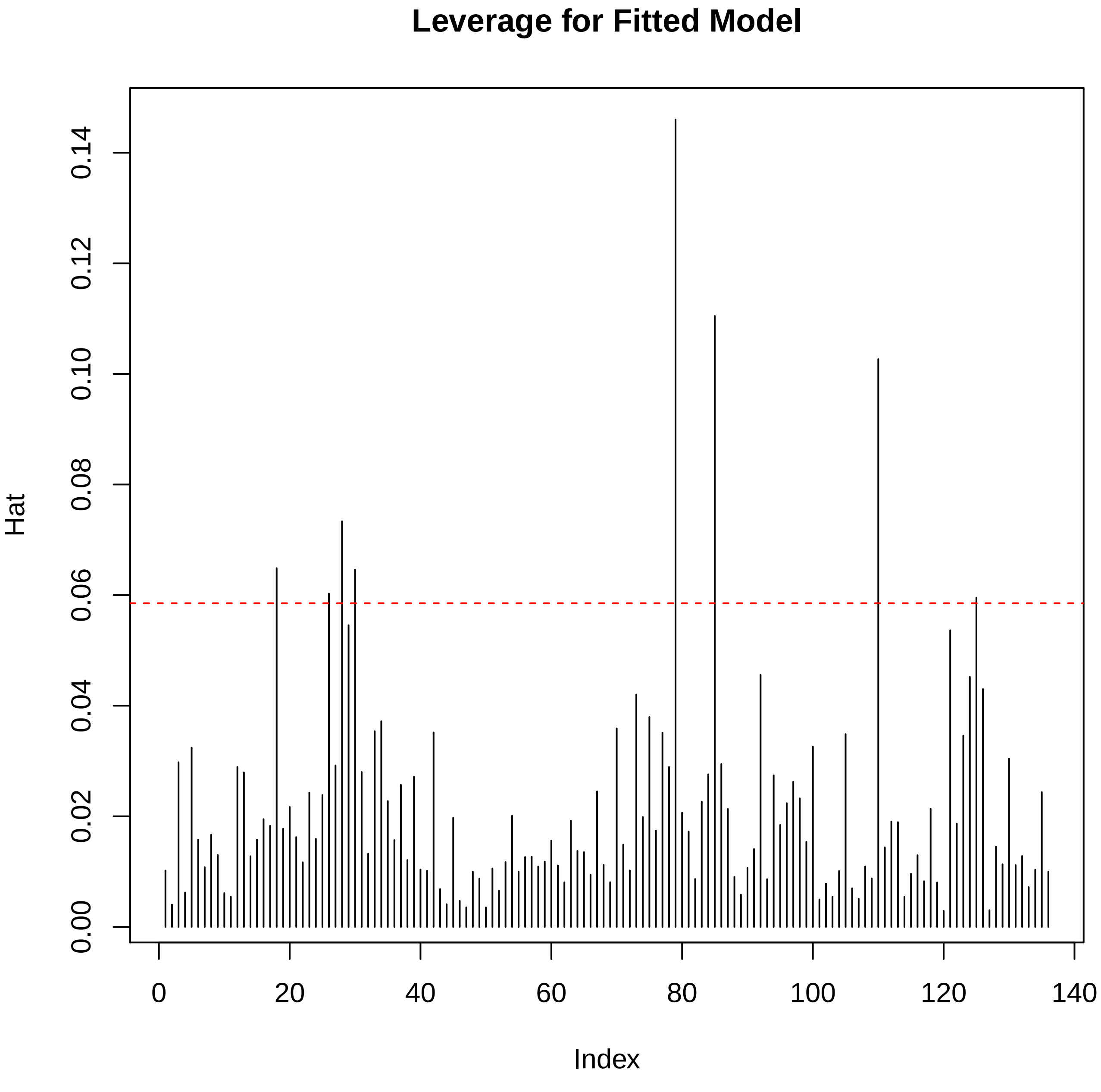
Leverage for Fitted Model
Hat
0.00
0.02
0.04
0.06
0.08
0.10
0.12
0.14
Index
0
20
40
60
80
100
120
140
